# Exploitation of Diversity within Crops—the Key to Disease Tolerance?

**DOI:** 10.3389/fpls.2016.00665

**Published:** 2016-05-20

**Authors:** Adrian C. Newton

**Affiliations:** Cell and Molecular Sciences, James Hutton InstituteDundee, Scotland

**Keywords:** tolerance, yield loss, mixtures, monocultures, asymptomatic, disease, traits

## Abstract

Tolerance, defined as the ability of a crop to maintain yield in the presence of disease, is a difficult characteristic to measure, and its component traits are generally undefined. It has been studied as a characteristic of plant genotypes grown singly or in monoculture crop stands. However, it is similarly valid as a characteristic of ecosystems, or mixtures / inter-cropping in crops and this paper seeks to evaluate theoretical and practical aspects of tolerance in this context. Focusing on cereals and fungal pathogens, consideration is given to the process of yield formation, the impact of disease on yield, and how tolerance might be assessed in monocultures. Variation in tolerance traits in monocultures and how such plants might interact in mixtures is considered; specifically the expression of tolerance in mixtures and how plants with contrasting tolerance traits in monocultures combine. Having focused on disease, further consideration is given to the impact of and on other microbial species in the crop environment. Finally the practical approaches that could be adopted to identify and assess the main traits responsible for expressing tolerance are addressed. These focus on the dynamic nature of plant–plant and plant-microbe interactions particularly in response to both biotic and abiotic stress out with the range of optimal or normal crop evaluation environments. It is proposed that by using more extreme factor parameter values in mixed crop evaluation environments the key traits affecting tolerance will be identified.

## Introduction

Tolerance of disease may be defined as the ability of a crop to maintain yield in the presence of disease (Schafer, 1971; Bingham and Newton, 2009). That crops differ in their disease tolerance has been recognized for many years, but recently there has been renewed interest in identifying the traits and associated mechanisms that underlie these differences so that tolerance may be increased through crop improvement or agronomic practice (Parker et al., 2004; Bingham and Topp, 2009; Bingham et al., 2009; Bancal et al., 2015). Several factors have prompted this interest. Focusing on cereals, only partial host resistance is available for many important plant pathogens and evolution of pathogen insensitivity to fungicides erodes their effectiveness in disease control. Improved tolerance is thus viewed as a complimentary approach to disease management because it will minimize the impact of disease on yield in cases where epidemics cannot be controlled fully by resistance mechanisms or the application of fungicides. Tolerance is also considered to be a potentially durable form of disease management, unlike disease resistance and fungicides, since it is expected to place little or no selection pressure for resistance on pathogen populations.

Box 1 **Terminology**

*Disease:* The visual expression of microbial challenge to plants, i.e., the symptoms. Symptoms can be varied but often show a high degree of correlation with loss of green leaf area. Disease does not necessarily equate to microbial infection as infection is often symptomless.

*(Plant) trait:* A genetically determined characteristic or condition. (Based on The American Heritage® Science Dictionary Copyright ©2002, published by Houghton Mifflin.) Traits may be physical, such as plant height or leaf shape, or they may be behavioral, such as rapid growth and late-flowering, or biochemical such as a disease resistance and salt tolerance. Traits typically result from the combined action of several genes, though some traits are expressed by a single gene.

*Trait modifier:* Any environmental or genetic factor that influences the expression of a trait, for example temperature or agrochemical treatment.

*Trait complex:* A set of interacting traits that can be measured together in one or more ways. A good example of a trait complex (/ complex trait) is yield that could be measured simply by weight, or divided into sub-classes and weighed etc.

To date, traits (see Terminology in Box 1) and mechanisms that confer disease tolerance have been investigated for crops grown as monocultures of relatively uniform, genetically similar individuals. Tolerance can be studied at the organ or plant level too, but the focus here will remain the crop as a primary aim of this paper is to identify the traits that are expressed in the field crop context and not necessarily in other contexts. With respect to disease this may be critically-important as disease epidemics are a constant threat in genetically uniform crops (Finckh et al., 2000), but in climax ecosystems they are the exception. Increasing the genetic diversity within cropping systems through the use of variety or species mixtures offers a number of potential advantages not only in terms of restricting disease development, but also increasing yield stability and resilience to abiotic stress and delivering other ecosystem services including greater biodiversity (Schöb et al., 2015). Plant-plant interactions are more complex in genetically diverse populations and may involve replacement, facilitation and niche complementarity effects (Brooker et al., 2016). Little consideration has been given to the nature of disease tolerance in mixtures and thus it is not known whether the methods for quantifying tolerance and identifying influential morphological and physiological traits that have been developed for monocultures are appropriate for use in variety or species mixtures. In this paper the concept of disease tolerance is reviewed briefly as developed for genetically uniform crops and the nature of plant–plant interactions in genetically diverse populations, before exploring whether putative tolerance traits identified for monocultures can be exploited in mixtures.

## Yield formation and the impact of disease

Crop yield (Y) can be quantified in terms of the amount of photosynthetically active radiation (PAR) incident upon the crop (I), the fraction of the PAR that is intercepted by green tissue (f), the efficiency with which the energy from PAR is converted into dry matter radiation use efficiency (RUE) and the fraction of the total above ground biomass that is allocated to the harvested parts the harvest index (HI; Monteith, 1977; Reynolds et al., 2005; Bingham et al., 2009; Murchie et al., 2009).

$$\begin{array}{cc} \text{Y}=\text{I}\times \text{f}\times \text{RUE}\times \text{HI} & \;(1) \end{array}$$

Equation (1) has been used as the basis for analysing variation in yield in response to geographical location, seasonal variations in weather, abiotic and biotic stresses including fungal disease (Johnson, 1987; Waggoner, 1990; Gaunt, 1995; Paveley et al., 2001; Bingham et al., 2007a,b). Disease may reduce crop growth by reducing radiation interception and RUE (Johnson, 1987; Bingham and Topp, 2009), although for a number of pathosystems the major effect appears to be the reduction in radiation interception with smaller or negligible effects observed on RUE (Rabbinge et al., 1985; Van Oijen, 1990; Robert et al., 2004). Depending on the timing of the disease epidemic, radiation interception by healthy (green) tissue can be reduced by effects of pathogens on leaf growth or healthy leaf area duration.

Disease, i.e., symptoms (see Terminology in Box 1), does not necessarily equate to microbial infection as infection is often symptomless. Infection can result in several types of trophic relationships including beneficial or mutualistic relationships such as rhizobium–legume interactions. In this paper the focus is mostly on microbes described loosely as pathogens from an anthropocentric perspective because they produce symptoms. However, used in this context the term pathogen is misleading as it obscures two essential attributes of these plant-microbe interactions that are relevant to consideration of tolerance. Firstly, the interactions can be either parasitic or pathogenic and secondly, they can transition between these states (Newton et al., 2010b). Indeed they can transition with the mutualistic state too and this will be considered later. Examples of diseases resulting from infection by microbes that are normally in the parasitic state are the cereal rusts and powdery mildews, where damage is caused primarily by loss of assimilates to the fungus and loss of active green leaf area from fungal structures mostly associated with sporulation. Also described as biotrophic interactions, the assimilate drain can be an active process where the fungus manipulates host metabolism and the net result is accelerated leaf senescence. Examples of pathogenic interactions are diseases caused by *Botrytis cinerea* and *Sclerotinia sclerotiorum*. Also described as necrotrophs, toxins are used to actively kill host tissue to render it accessible as a substrate for microbial growth. Some microbes may occupy either of these states (or the mutualistic state) at different times in their lifecycle with respect to the host plant and are often described as hemi-biotrophs. *Ramularia collo-cygni* and *Rhynchosporium commune* on barley are good examples of microbes that transition between states during their life cycle. They grow asymptomatically within tissues for considerable periods but following certain triggers they produce toxins and visible symptoms (Newton et al., 2010b). In some, but not all, pathosystems changes in host metabolism can precede the development of visible symptoms (Scholes and Rolfe, 2009). At present it is not known whether the asymptomatic infection incurs a metabolic cost to the plant, but clearly whether this occurs and when the transition to the symptomatic state takes place will have implications for yield formation as well as the measurement of tolerance. This is because biotrophic and necrotrophic infection can lead to a range physiological changes related to leaf carbon metabolism, including increased rates of respiration, reduced rates of net photosynthesis, alterations in stomatal conductance and chlorophyll concentrations and reductions in the amounts and activities of Calvin-Benson cycle enzymes (Roberts and Walters, 1988; Murray and Walters, 1992; Prats et al., 2006).

Tissue death associated with lesion development by either necrotrophic or hemi-biotrophic pathogens results in a loss of green area and some shrinkage of the leaf surface. The parasitic interactions too lead to premature loss of green leaf area. As symptomatic tissue continues to intercept and absorb a significant fraction of the incident PAR, the amount of radiation intercepted by healthy tissue is correspondingly reduced. The effects of disease on carbon metabolism described above can also reduce the efficiency of conversion of energy from absorbed PAR into dry matter production. In crop growth analysis, RUE is usually quantified from the slope of the relationship between above ground biomass gain and cumulative radiation interception (Bingham et al., 2007a,b). Thus, any effect of disease observed on RUE will be the net outcome of its effects on canopy photosynthesis, respiration and biomass partitioning between roots and shoot.

The impact of reductions in radiation interception and RUE on yield will depend on how disease influences assimilate partitioning and the source-sink balance of the crop. HI is measured at harvest as the final expression of dry matter allocation, but is determined over the course of the crop life cycle. It is influenced by the effects of genotype, crop management and environmental factors (including disease) on the relative growth of photosynthesizing (source) and yield bearing (sink) organs and the deposition and subsequent remobilization of temporary storage reserves. In determinate crops such as wheat and barley, vegetative growth prior to flowering determines the size of canopy produced and the number and potential storage capacity of grains. The number of grains is determined by the production and survival of tillers and the production, survival and fertilization of spikelets or florets. The potential storage capacity of grains has been related to the size of the carpel at flowering and the number of endosperm cells produced shortly after fertilization. The periods of tiller and spikelet/floret mortality and differentiation and growth of the carpel coincide with the phase of rapid stem extension and there is evidence that these processes are influenced by availability of assimilate during this time. Stem water soluble carbohydrate reserves are also deposited as the stem extends. Timing is critical. Thus, early disease epidemics which develop prior to flowering can simultaneously restrict both source (canopy healthy area and deposition of stem soluble carbohydrate reserves) and grain sink capacity (numbers and storage capacity of grains). Late disease epidemics, on the other hand, restrict assimilate availability for grain filling by reducing canopy healthy area and post-flowering photosynthesis. The negative effects of late disease on grain filling may be buffered by the remobilization of temporary storage reserves.

Not all periods of the crop lifecycle are equally sensitive to abiotic or biotic stress (Ney et al., 2013). In cereals, stress that develops around flowering can be especially damaging to yield because of the irreversible reduction in grain sink capacity that can occur. For example in maize, water stress at flowering can result in the abortion of embryos and a permanent reduction in kernel number. The effect is associated with a reduction in photo-assimilate supply to the ear and can be prevented, in part, by the exogenous supply of sucrose (Zinselmeier et al., 1999). In summary, differences in these mechanisms that together affect yield will have different implications for tolerance to disease.

## Disease tolerance in monocultures

Traits that enable radiation interception, RUE and dry matter partitioning to be maintained in spite of disease will minimize yield loss and hence confer tolerance of disease. Therefore, there are many potential tolerance traits that may operate at a range of organizational levels from the organ through to the crop (Ney et al., 2013). In addition, whether or not a particular trait or trait combination is identified as contributing to tolerance will depend on the techniques used to quantify disease and its relationship with yield (Bingham et al., 2009). Candidate traits conferring tolerance and the issues surrounding the measurement of tolerance have been discussed in detail elsewhere in the context of crop monocultures (Bingham and Newton, 2009; Bingham et al., 2009; Ney et al., 2013) and thus only a brief overview is given here.

The impact of fungal infection on net photosynthetic rates within an infected leaf can vary with both the pathosystem and location of the tissue relative to the disease lesion. There is some evidence of an increase in rate in symptomless regions of diseased leaves (Last, 1963; Habeshaw, 1984), although a reduction is a more common observation (Martin, 1986; Bastiaans, 1991; Scholes and Rolfe, 1995). Similarly, increased rates of photosynthesis in non-infected leaves of diseased plants have also been reported (Roberts and Walters, 1986; Rooney and Hoad, 1989; Murray and Walters, 1992). There have been few attempts to quantify the extent of intra-specific variation in these responses, although there is some limited evidence that intra-specific variation exists in the response of wheat leaves to septoria leaf blotch (Zuckerman et al., 1997). An increase in photosynthetic rate in apparently healthy tissue, made in response to the development of disease elsewhere on the plant, could lead to tolerance by compensating for the loss of healthy tissue and thus maintaining yield. However, for any particular pathosystem it would need to be established that the increase is indeed compensatory and results in carbon fixation that is used to support yield formation rather than just the biosynthesis of defense compounds (Tiffin, 2000). Morphological plasticity is another mechanism by which plants might restore photosynthetic capacity in response to defoliation. Although most widely documented for plants defoliated by herbivory, reductions in the allocation of biomass to root growth relative to shoots and an increase in leaf area ratio have also been observed in several pathosystems involving foliar disease (Walters and Ayres, 1981; Paul and Ayres, 1986; Rooney and Hoad, 1989).

It has been postulated that cereals whose grain storage capacity (sink capacity) is small relative to their ability to supply grains with photosynthate during grain filling (source capacity) will be relatively tolerant of post-flowering disease (Gaunt, 1995; Bingham et al., 2009). There is evidence that the yield of many crops is sink-limited (Borrás et al., 2004), but that the extent of the source-sink imbalance varies widely between sites and years (Bingham et al., 2007a,b). This would suggest that tolerance of post-flowering disease might also vary widely between crops. Carbon assimilates for grain filling come from concurrent photosynthesis and the remobilization of temporary storage reserves, although the contribution of the latter differs between species. Intra-specific variation in the concentration of water soluble carbohydrate reserves in wheat has been reported, prompting speculation that genotypes with large reserves will be more tolerant of disease (Foulkes et al., 2002). However, direct evidence to support this has not yet been found.

Modeling of canopy photosynthesis in diseased crops suggests that canopy size and architecture are traits that may influence tolerance (Bingham and Topp, 2009). Large canopies and canopies with a relatively high light extinction coefficient were found to be relatively more tolerant of disease especially if disease was located in the lower canopy. This is because in those canopies most of the incident light is intercepted by the upper leaf layers and the lower-most leaves contribute little to canopy photosynthesis (Bingham and Topp, 2009).

## Quantifying tolerance variation

As many of the potential mechanisms conferring tolerance operate at the canopy level, measurements are generally made in field experiments (Parker et al., 2004; Foulkes et al., 2006; Bancal et al., 2015). Achieving equivalent disease severity across a range of genotypes is almost impossible under field conditions and so an approach is adopted in which disease severity is varied over a defined range by inoculation or by using fungicides as necessary. Tolerance can then be quantified as the change in yield per unit change in disease severity. The most common measurement of disease severity has been the Area Under the Disease Progress Curve (AUDPC) which integrates disease severity over time (Kramer et al., 1980; Newton et al., 1998). However, measurements of AUDPC provide no indication of the amount of healthy tissue remaining. As the relationship between canopy area and radiation interception is non-linear, variation in canopy size and hence residual green (healthy) area can have an appreciable effect on the reduction in crop growth or yield under a given disease severity (Bingham and Topp, 2009). Canopy growth is sensitive to variations in soil, climatic and crop management factors and this may contribute to the large variation observed in AUDPC-yield loss relationships and designations of tolerance for varieties across sites and seasons (Kramer et al., 1980; Johnson, 1987; Waggoner and Berger, 1987; Newton et al., 1998, 2000). In order to minimize this problem in wheat and provide a more robust estimate of genotypic variation in tolerance across environments, post-anthesis healthy area duration has been used as a surrogate for disease severity as it links more directly with radiation capture (Parker et al., 2004; Ney et al., 2013).

Characteristics of plant-microbial interactions and host traits that might influence the designation of tolerance by modifying disease-yield loss relationships are categorized in a hierarchical way in Table 1: (1) asymptomatic and symptomatic microbial challenges resulting in differential effects on yield loss relationships by inoculum pressure / pathogen challenge and disease symptom expression variability; (2) yield compensation, facilitation and competition responses to disease and plant developmental responses; (3) protocol effects including the effects carried over from previous crop treatments, seed health or environments (epi-genetic) and of fungicide mode-of-action types favoring germplasm differentially either through direct physiological responses or differential effects on asymptomatic microbial infections / challenges. Most of these traits also show interaction with: (4) plant developmental stage, nutrients, environment / weather, abiotic stress etc., some of which might be expressed in terms of yield sensitivity, for example response to site fertility affecting varieties differentially (Finlay and Wilkinson, 1963).

**Table 1:** **Groupings and types of mechanisms or factors that might impact disease / yield loss relationships in plant communities**.

**Group**	**Factors and mechanism**	**Impact on yield**
(1a) Microbial asymptomatic infection	Parasitic	−−
	Mutualistic/beneficial	−/+
(1b) Pathogen microbial challenge	Hypersensitive resistance (HR)	−−−
	Partial and non-HR resistance	−−
	Susceptibility	−−−
(2) Developmental response to plant or microbial interaction / challenge	Compensation growth	+
	Facilitation response	+
	Competition response	−/+
(3) Protocol effects	Previous crop legacies (e.g., microbial inoculum / anti-microbial substances	−−/+
	Plant physiological legacies (vigor etc.)	−/+
	Epigenetic legacies on plant physiology / gene expression	+
	Direct fungicide / agronomic treatment effects on plant physiology	−/+
	Indirect fungicide / agronomic treatment effects on microbial challenges	−/+
	Assessment methodologies	−/+
(4) Environmental modifiers of 1–3 above	Nutrient availability	−−
	Weather / climate	−−
	Abiotic stress (cold / drought / salt etc.)	−−
	Soil (root stress, nutrient availability etc.)	−−

Impact on yield scale from very negative to positive (− − −, − −, −, +) is arbitrary and dependent on appropriate measurement method for validation.

As disease tolerance is defined and measured in terms of visible disease severity or a surrogate, the effects of asymptomatic microbial infection on plant growth and yield are particularly important. These may be classified as parasitic, mutualistic/beneficial or pathogenic and each state may be associated with different physiological interactions and therefore effects on host metabolic processes resulting in different effects on tolerance. Furthermore, for many plant-microbe interactions these interactions are dynamic and transition through a lifecycle. Hence these are divided into: (1a) asymptomatic challenge, either parasitic or mutualistic / beneficial, and (1b) symptomatic which is largely synonymous with pathogenic challenges (Table 1). The latter result in either hypersensitive resistance with minimal symptoms, some form of partial or non-hypersensitive resistance, or susceptibility. In addition to visual and other biomass assessment methods, defining molecular mechanism and specific gene expression profiling will be highly informative. The different response types will differ in expression levels of some pathways, for example lower defense pathway expression in non-pathogens. Equating these to energy or assimilate cost would have great potential for correlation with yield response. In molecular terms pathogen and non-pathogen responses are usefully classified as Pathogen-Associated Molecular Patterns (PAMPS) and Microbe-Associated Molecular Patterns (MAMPS) respectively (Newman et al., 2013). However, within each group, inoculum pressure will show its own dynamic interaction and is affected by the ability of each host to support sporulation. Sporulation can occur whether visible symptoms are present or not (Newton et al., 2010b) though it is likely to be greater in pathogenic interactions. Some varieties are likely to be carrying different microbial loads, not necessarily pathogens though. For example, the old cultivar Igri carries a different microbial population from most other winter barleys (Gravouil, 2012). Germplasm identified with traits that affect the potential untreated yield loss may be due to fewer biotic interactions that cause induction of defense when this is not necessary, or selection for detrimental rather than beneficial microbial phylloplane populations.

The consequences of the microbial interactions are expressed in the second group (Table 1) that impacts yield loss relationships, i.e., the developmental response to plant or microbial interaction or challenge. Whilst these processes operate in monocultures (i.e., self-competition), their importance will be discussed more in the context of diversity.

Many apparent tolerance traits are responses to particular attributes of the experimental or growing protocols used, our third group (Table 1). These need some careful consideration if methodologies for detecting tolerance are to be developed. The rationale for good crop rotation practice is to maintain soil health described in terms of soil physical and microbial structure, nutrients and pathogens. These can include practices that induce shifts in the microbial spectrum including promotion of root exudates with anti-microbial properties. However, soil microbes are crucial not only to soil processes that then affect plant growth, but also many induce plant responses directly. The most studied are classed as Induced Systemic Resistance (ISR) whereby microbes such as *Pseudomonas* species induce specific defense pathways that make above-ground parts of the plant resistant to many pathogens (Kuć, 2001). Induction of resistance has energetic cost that must be considered in the overall defense strategy of the plant and will therefore impact yield loss relationships. A good rotation keeps all these things in balance or within an acceptable range. However, when they are out of balance tolerance traits may be easier to identify (see below).

Another possible factor that may influence tolerance is a plant physiological legacy such as vigor. This could be simply related to seed resources such as endosperm size or composition. They could be also epigenetic legacies on plant physiology or gene expression and evidence is accumulating rapidly that these may be very common (Walters and Paterson, 2012; Pastor et al., 2013). The mechanisms are beginning to be identified together with the genetic loci controlling them (Luna et al., 2014). As these genes respond to environmental triggers, demonstrating their effect and relationship to tolerance is difficult but potentially very important both for agronomic management and financial benefit.

Assessing the effects of agronomic treatments such as the application of fungicides is often not as simple as determining the reduction in pathogens and subsequent disease. A fungicide application has effects on plants due to (1) the physical spray / formulation / adjuvant composition, and (2) mode of action, and each of these will impact both (a) the microbial population composition and (b) plant metabolic processes. The net result again affects apparent tolerance characteristics. For example prothioconazole and pyraclostobin increase grain number in spring barley in the absence of disease whereas chlorothalonil did not (Bingham et al., 2014). Biostimulants, whether specific products, the indirect effects of resistance elicitors or indirect effects of certain fungicide modes of action are even more likely to impact yield loss relationships and are another example of where molecular analyses of gene expression could be very helpful in understanding mechanisms (Lyon et al., 2014).

The fourth group (Table 1) are the modifiers of tolerance such as nutrient availability, the day-to-day weather, the general climate, abiotic stress such as cold, drought, salination, temperature shocks, wind, soil physical characteristics as well as the microbial composition referenced above causing root physical stress and affecting water and nutrient availability. The effects of all such factors can be profiled in many ways, not least gene expression. Wind, rain and other touch treatments for example, affect overall plant growth form and health and subsequently the plant's ability to respond to other challenges (Braam and Davis, 1990). There are many common stress-response genes and biochemical pathways and these are key to what might describe as healthy or normal plants (Newton et al., 2012b).

The importance of understanding what mechanisms are operating in plant-microbial interactions is to identify whether consequential changes in yield will affect visible disease symptoms and therefore the classical definition of tolerance.

## Plant–plant and plant–pathogen interactions in mixed plant populations

Clearly different cultivars can be classified as having different expressions of the factors and mechanisms that affect tolerance. Therefore, their accurate and appropriate assessment is necessary to determine whether their combined expression in mixtures is additive or synergistic. The component combinations that contribute most beneficially to tolerance in mixtures can be dissected-out. Facilitative plant–plant interactions are “positive, non-trophic interactions that occur between physiologically independent plants and that are mediated through changes in the abiotic environment or through other organisms” (Brooker et al., 2008). It is widely recognized and demonstrated that heterogeneous plant communities produce more total biomass than monocultures (Newton et al., 2009; Schöb et al., 2015). The interaction of two or more crop species growing together and co-existing for a time can result in more efficient resource use through niche differentiation and complementarity. This reduces negative competitive interactions through reduced niche overlap but also enables enhanced resource availability through direct facilitation, for example the secretion by some crop species of substances such as organic acids and phosphatases to increase P availability in acidic soils or N transfer from nitrogen-fixing legumes to companion species (summarized from Brooker et al., 2016). There can be more general effects too such as hydraulic lift causing increased water availability to all the plant community (Prieto et al., 2012). Brooker et al. (2016) also cite pollinator attraction and protection from pests and similar effects below-ground through increasing plant biomass or diversity enhancing the density or diversity of beneficial soil microbes.

In Table 1 the interactions are classified as compensation, facilitation or competition but microbes are also a component of all these interactions, be they in the rhizosphere or the phylosphere. The dynamics of pathogen populations and heterogeneous plants have been investigated in many studies and often characterized by population modulating characteristics. One of the best-known benefits resulting from enhanced niche complementarity through indirect facilitation is disease and pest control. The diverse components within the crop contribute in several ways to reducing overall pest and disease incidence, specifically (1) dilution of susceptible individuals or preferred hosts, (2) the barrier effect of resistant individuals, (3) induction of resistance in individuals neighboring infected plants (Chin and Wolfe, 1984), (4) changes in vegetation structure and microclimate affecting infection processes and (5) providing a more heterogeneous resource supply that supports a higher abundance and diversity of natural enemies of crop pests (i.e., associational resistance; Gunton, 2011; Letourneau et al., 2011). These processes operate at both inter- and intra-specific levels (Newton et al., 2009; Kiær et al., 2012). The first two processes are physical spatial effects whilst the others are physiological and biochemical effects and are dependent on the challenging organism's mode of pathogenicity or parasitology, population structure, plant architecture, development stage and physiology, and of course many environmental variables. Furthermore, where defense mechanism are induced there can be a metabolic cost so the trade-off against potential loss must be positive. Such effects are compounded in polycyclic diseases when pathogen inoculum pressure is reduced at each cycle. Such pest and disease resistance effects are examples of facilitation. However, these effects on disease are most obvious when there is a moderate pathogen challenge on the crop because they can be swamped by too much inoculum (Newton et al., 2002).

## Disease tolerance in mixtures

Few attempts have been made to quantify the contribution of disease tolerance *per se* to the productivity of crop mixtures. In principle, individual genotypes in mixed populations might differ in their inherent tolerance via mechanisms discussed above that operate at the organ and plant level, although the expression of tolerance may conceivably be modified by external factors such as nutrition, solar radiation and plant–plant interactions (Table 1). If it is assumed that external factors have a minor influence or that each genotype is affected equally, then the tolerance of the mixture would be expected to be the same as the average of the tolerance of the individual components. For traits that operate at the crop level, on the other hand, such as canopy size and architecture (Ney et al., 2013), their influence on disease tolerance of the mixture will depend on the interactions between individuals and the spatial arrangements of leaves and disease within the canopy (Bingham and Topp, 2009). Where genotypes differ in their disease resistance, if the more susceptible genotypes have their leaves positioned lower in the canopy than the resistant ones, the impact of disease on canopy photosynthesis will be minimized and tolerance favored. The converse would be the case if the susceptible genotypes are the tallest and disease epidemics develop in the upper canopy.

Further, in mixed populations of plants with differing disease resistance, negative competitive interactions are likely to occur as a result of niche overlap. Here disease developing on one or more components could shift the competitive balance in favor of the non-diseased components leading to stability of productivity of the population. If this is measured in terms of biomass production or yield per unit of disease over time it can be viewed as tolerance and would be equivalent to compensatory adjustments in assimilation or growth of new organs in a monoculture. In this case tolerance of the population is not dependent on maximizing tolerance of the individual genotypes within the mixture. Indeed if the dominant genotype is disease tolerant, then competition with other components may be maintained in spite of the disease and thus adjustments in growth of subordinate components reduced. The overall effect, however, would be one of tolerance within the mixture.

This concept begs the question—is there any value in seeking to maximize the tolerance of individual genotypes, if tolerance can be achieved with mixtures of genotypes with contrasting/complimentary disease resistance? In other words, tolerance in mixed populations comprises an additional set of traits and mechanisms from tolerance in self-populations. Other factors must be considered when trying to answer this question. The extent of the tolerance in a mixture will depend on the capacity of subordinate genotypes to increase their yield. However, a relief of competition and increase in resource capture by subordinate genotypes may not necessarily lead to an equivalent increase in yield if the plant is sink-limited and at a developmental stage at which it cannot increase it's sink capacity in response to the increased resource availability. Potentially this is likely to be more of an issue when the mixed populations are composed of different species with contrasting resource use efficiencies in their formation of yield and the economic value of their harvested parts, as may be the case in some intercrops. Thus, protecting the yield of the most resource efficient and highest value component of the mixture through effective disease resistance and tolerance of the individual component may be more beneficial than relying on partial compensation for yield loss to disease within the mixture from other less efficient and lower value components. Similarly, if the yield advantage of a mixture in the absence of disease is dependent on facilitation mechanisms there may be merit in protecting the facilitator component by maximizing its individual disease tolerance or resistance so that the facilitation is sustained.

Yield loss relationships tend to fit a range of regression relationships that may compound multiple simultaneous relationships, some of which change behavior upon attaining thresholds. Mechanistically this is likely as inoculum-disease relationships often have such thresholds, classically expressed in quorum-sensing with bacterial diseases but expressed more incrementally in fungal diseases. The plant defense responses similarly have thresholds that must be exceeded before, for example, cell death processes are triggered as these are irreversible and costly to the plant. This may be reflected in the high cost of powdery mildew resistance caused by *Blumeria graminis* f.sp. *hordei* and conferred by the *mlo* gene in barley as this is characterized by very early or fast recognition and response from a mutant regulatory gene (Piffanelli et al., 2002). Major gene resistance to Septoria Leaf Blight caused by *Zymoseptoria tritici* in wheat is similarly costly when effective and may have a mechanistic explanation also (Brown, 2002). In both cases these represent non-tolerance traits analogous to trait over-expression.

In mixtures losses in more diseased components will be compensated for partially by the less damaged components. However, other interactions between components may be contributing more to the mixture advantage through enhanced resource capture rather than compensating for loss. This is often true as even when there is a strong correlation between component number in the mixture and disease reduction that is reflected in yield, the same correlation is clear in the absence of disease suggesting that this the non-disease control interactions are dominant (Newton et al., 1997). Therefore, mixture advantage would be expected to be greater if this is the case when surplus resources are available such as under higher fertilizer rates. This is what is often found in practice (Newton et al., 2012a). The candidate traits are those that enable more of the available resources to be captured and/or for more time. This is clearly shown when contrasting canopy types are combined such as those expressing either, neither or both of the two common dwarfing genes in spring barley. Whereas, normally combinations of elite spring barley genotypes show small gains in the order of 0–3% above the mean of their components, 10% was achieved with three-component mixtures of these contrasting canopy type components (Newton et al., 2004).

## Exploitation of diversity

Crops are communities of plants bred and grown in self-competition in a monoculture crop typical of much intensive agriculture. The fundamental approach is to express all desirable traits to their optimum state to produce ever improving yields. Diversity is exploited in this process, but only by selecting strong or extremes of desirable trait expressions. This approach is very successful for many traits so there is a tendency to assume that it will be successful in general and therefore applied to all traits. However, this may be a fundamentally flawed assumption for other traits, especially plant interactions with other complex communities of other organisms, particularly pathogen interactions but microbial interactions in general for the reasons outlined above.

**Figure 1:**
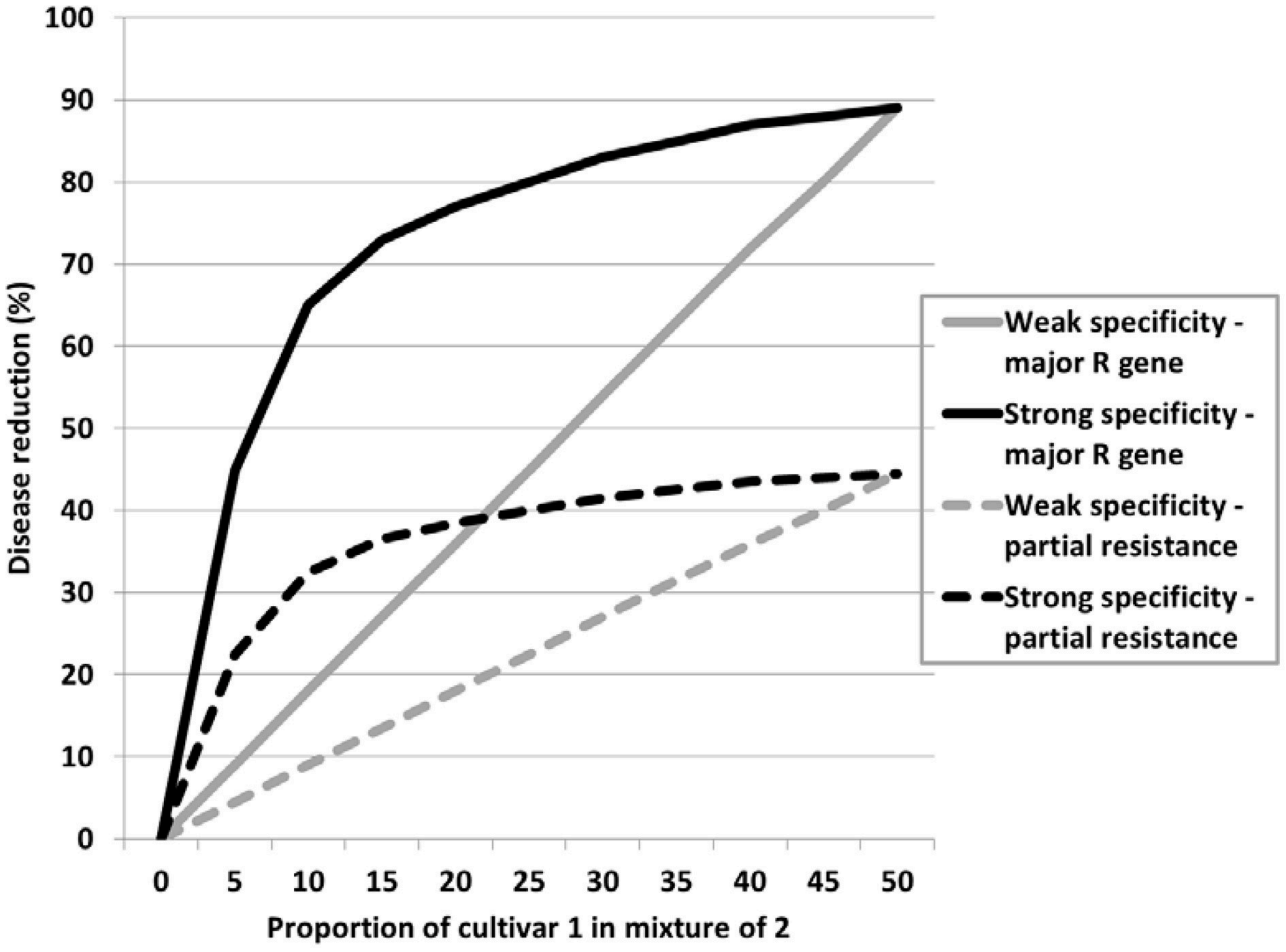
**Trait strength and size relationships in mixtures**.

Disease-reducing traits, whether specific resistance, non-specific resistance, or factors that affect infection and subsequent disease development, may have varying levels of expression. These can be classified as strong or weak expression such as classical major gene resistance and partial resistance to cereal rusts respectively. In a cultivar mixture the effects of either may increase with mixture proportion, but the maximum effect of the strong expression trait will be greater than the same proportion of the weak expression trait. These are represented by the straight diagonal lines in Figure 1. However, this also assumes specialism, i.e., each component cultivar expresses effective resistance only against a proportion of the pathogen population. Where this specificity is strong a small proportion of either cultivar will have a disproportionately large effect as represented by the curved lines in Figure 1. This is supported by both experimental data (Newton and Guy, unpublished data) and modeling (Mikaberidze et al., 2015). Generalizing this, if it is assumed that specificity strength is generally a measure of trait strength and major gene or partial resistance equates to the magnitude of the trait expression overall (size), then this can be applied to other traits to help design mixtures and predict outcomes. Thus, strong traits can be exploited in small proportions whatever their overall or maximum expression might be. Strong can be interpreted also as traits with contrasting expressions. Thus, canopy types such as tall, semi-prostrate, erectoid and double-dwarf conferred by the combinations of two dwarfing genes referred to in the last section are very strong and contrasting trait expressions and indeed combinations have strong positive interactions greater than the weighted means of their components in terms of yield benefit (Newton et al., 2004).

Before moving on to discuss these community plant-pathogen interactions more, It should be acknowledge also that the ideal trait assembly forming a very superior crop plant is rare and that by assembling different crop cultivars with different and complementary traits, overall crop performance can be enhanced. Elite germplasm developed and exploited under optimal agronomic and environmental conditions generally offers few opportunities for exploiting complementarity, be it through competition or facilitation, as most traits have very similar expressions. In any single year and on individual sites, single cultivars are likely to be the top performers, but it is unlikely that any one cultivar will be top on all sites and in all years. Under real farm conditions that are seldom uniformly optimal and across the years, heterogeneous assemblies of elite cultivars are likely to out-perform the mean of the components grown separately (Finckh et al., 2000; Newton et al., 2009; Kiær et al., 2012). However, the greater opportunities may come from associations with other crop species where many traits have strong or highly contrasting expressions and the opportunities for complementation are much greater.

## Crop diversity from the microbial perspective

Very little is known about the non-pathogenic microbial component of these heterogeneous plant communities in the phyllosphere, though it is known that they enhance microbial diversity in the rhizosphere (Johnson et al., 1992; Lawrence et al., 2012). Another dimension can be added to this, that of pathogen/parasite-non pathogen interactions as these represent a complex spectrum of interactions ranging from hyper-parasitism (Kiss, 1997) to mutualism where the disease is caused or exacerbated by a microbial complex (Newton and Toth, 1999). However, focusing on the plant response, whether beneficial resources are supplied or damage is caused, plants respond to enhance their fecundity in ecological terms, though this may be distorted in crops (Newton et al., 2010a).

The focus on defense against disease is often driven from a highly anthropocentric rather than ecological point-of-view. Disease is assumed to be caused by pathogens and a classical “arms race” approach is often used to describe defense strategies. However, understanding the nature of plant-microbial interactions in a more ecological framework often leads to a more sustainable “soft power” or diplomatic approach. Disease is simply a particular outcome of a plant-microbe interaction with specific spatial and temporal parameters. In an ecological context the same plant and microbe may also exhibit mutualistic or parasitic interaction at other times or places. Overall both plant and microbe are likely to benefit from their association, but at any one time the balance may be skewed strongly toward one or the other. Essentially the relationships between plants and microbes are dynamic. However, in the case of crop plants where the economic yield component has been greatly enhanced. This presents a large substrate to the microbial community with a narrow range of expressions of plant defense mechanisms which is normally to the microbe's advantage. Even then the association may be either pathogenic or parasitic depending whether the host is actively damaged using necrosis-inducing mechanisms such as Botrytis infection on lettuce, or simply drained of resources, the rust pathogens on cereals being a classic example of the latter (Browder, 1985). Pathogen communities often generate a reservoir of trait variation that can overcome plant defenses. However, a single genotype host generally has only a narrow range of expressions of defense and the only back-up defense is with replacement genotypes from the plant breeders. In a community of plants the back-up is in the plant community that is being constantly challenged and selected.

As plants in dynamic association deliver community benefits through competition and facilitation, so too microbes work in association to more effectively interact with their host. Examples of this are found in complex microbial infections where one organism may be the apparent “causal agent” but disease symptoms are the expression of several working together for mutual benefit, again through competition and facilitation (Dewey et al., 1999). Microbes also deliver benefits to the overall plant-microbial interaction for both partners through component dynamic mutualist-pathogen-parasite interactions, i.e., competition and facilitation (Newton et al., 2010b). Put these together and a complex web of interactions is assembled comprising many, varied and dynamic competition and facilitation relationships.

## Measuring tolerance in mixtures

Tolerance in mixtures is potentially more complex and uses different mechanisms compared with monocultures, thus the task of identifying the contribution of individual components to tolerance and their response to modifying factors represents as a considerable challenge. Nevertheless, a greater understanding of tolerance and its contribution to resource use efficiency and yield stability of mixtures would allow a more rational approach (greater element of crop system design) to exploitation of crop diversity in disease management.

Tolerance is the combination or sum of several traits and their combination in plant communities, so how should their importance be ranked and how can their parameter range be calibrated or profiled? Using molecular biology terminology, the best strategy might be to use knock-outs and/or over-expression of key traits of factors that influence them. Only when particular traits expressions are removed or exaggerated will their contribution to the composite tolerance trait be strongly expressed and measurable, i.e., when the system is out of balance or unstable.

An example of over-expression is the effect of inoculum pressure and fertilizer on tolerance designations in spring barley (Newton et al., 2000). It was not possible to identify barley genotypes that were consistently tolerant across all trial conditions. However, there was good agreement between the both low and high fertilizer conditions under high inoculum pressure and there was also good agreement between the low fertilizer conditions under both low and high inoculum pressure. There was also good agreement between high inoculum + high fertilizer and low inoculum + low fertilizer, in other words the more contrasting or over-/under-expression conditions resulted in stronger expression of the tolerance composite trait.

A second example involving inoculum pressure and tolerance is the effect on mixture efficacy. As noted above (Table 1), group 2 heterogeneous plant communities generally increase biomass production and decrease disease. Group 1b pathogenic and non-pathogenic biotic challenges balances the cost of defense with these interactions. However, whilst under high inoculum pressure mixtures consistently reduced relative disease less, an increased yield response did not necessarily follow (Newton et al., 2002). This is likely because the pathogen control effects in mixtures were not the dominant interaction leading to enhanced yield in these trials.

Designing “over-expression” and “knock-out” treatments that might be used to parameterize the expression of tolerance traits will be difficult from many points-of-view. The first will be designing the comparator. Although this should be “optimal” conditions, all conditions are in fact compromises and plants need to be exposed to a range of both biotic and abiotic conditions to grow “normally” and therefore arbitrary norms should be defined. Some parameters that might be manipulated experimentally could be over-expressed or strongly under-expressed / knocked-out, bearing in mind that they will likely have consequences for other parameters (Table 2). For example, providing a nutrient in excess or deficiency will likely affect uptake of other nutrients both directly and indirectly. Nevertheless, these conditions may help identify groups of germplasm with common trait expressions behaving similarly as potential component traits of tolerance in mixtures.

**Table 2:** **“Over-expression” and “knock-out” treatments that might be used to identify factors that affect tolerance traits in plant communities**.

**Trait group**	**“Knock-out”**	**“Over-expression”**	**Comparator**
Microbial challenge—airborne inoculum	Clean air; disinfected environment; inert microbe-free growing medium	Heavy / frequent inoculation; multiple species microbial challenges above- and below-ground, with pathogen / non-pathogen	“Optimal”^a^ controlled environment; “normal”^a^ field environment
Microbial challenge – waterborne inoculum	Clean water	High spore/mycelial concentration inoculation; multiple species microbial challenges above- and below-ground, with pathogen / non-pathogen	“Optimal”^a^ controlled environment with low inoculum treatment; “normal” field environment
Water	Drought	Waterlogging	Field capacity
Temperature	Low / high mean	Heat / cold shock	“Optimal” controlled environment
Nutrient	Series of single and multiple nutrient deficiencies	Series of single and multiple nutrients in excess	“Optimal” fertilizer
Crop protectants / stimulants	Range of fungicide modes of action	Resistance elicitors and biostimulants with and without pathogen challenge^b^	Standard crop agronomic protocol or clean environment
Light	Low level light; short daylength	High intensity, wavelength-specific treatments combinations; long daylength	“Optimal” light in controlled environment or field
Atmosphere	Low CO_2_ concentration	High CO_2_ concentration, high ozone concentration	“Normal” atmospheric composition

a Arbitrary comparison or reference level.

b Priming response only expressed with subsequent pathogen challenge.

**Figure 2:**
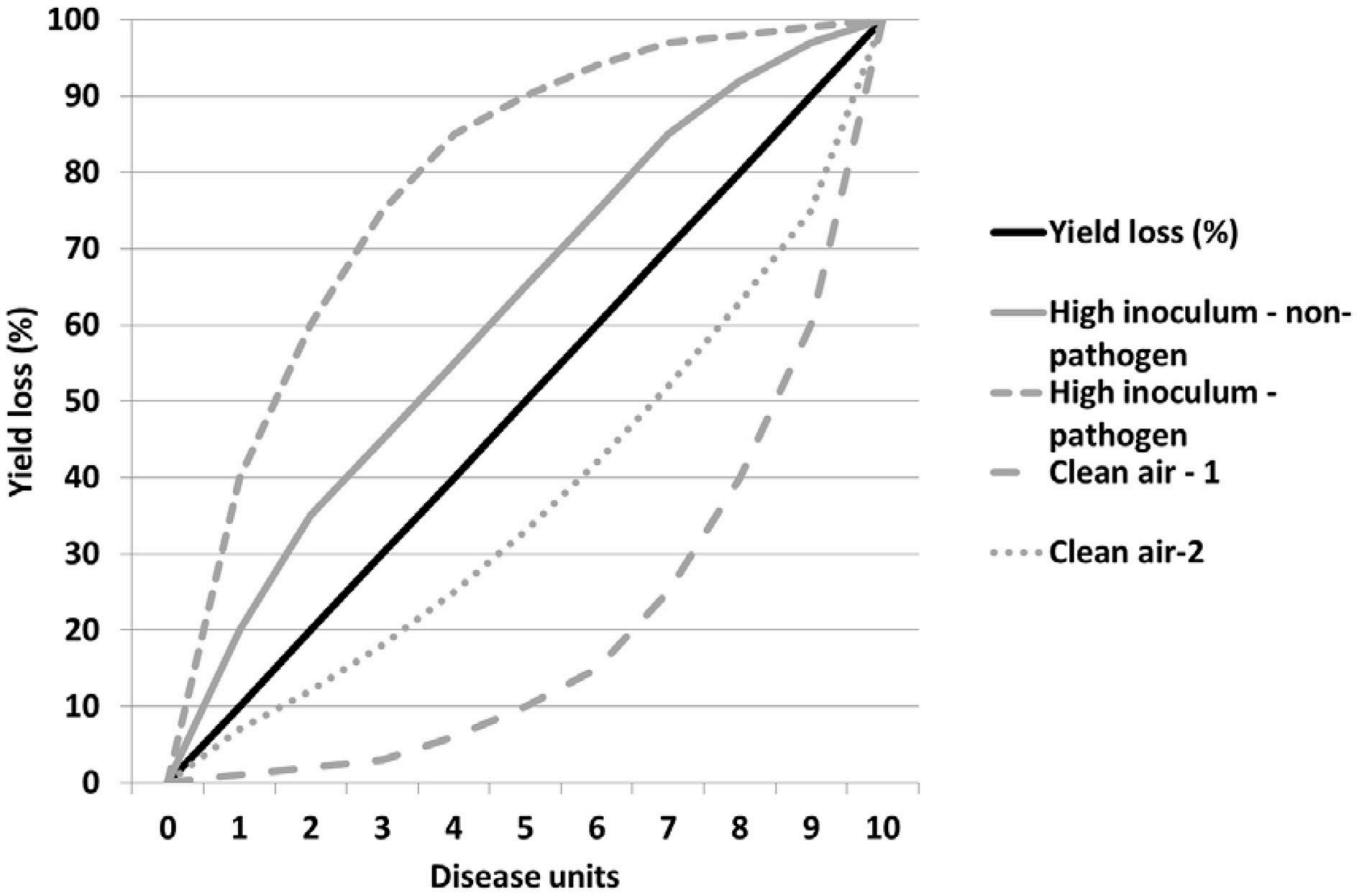
**Factors affecting the expression of yield loss and tolerance detection**.

The relationship between yield loss and disease may not be always linear, perhaps especially toward the extremes (Madden et al., 1981). Even if we assume it is, how knock-out and over-expression of influencing traits will affect this relationship may vary. Figure 2 shows how a regression might change its slope positively or negatively in response to heavy inoculum pressure or the absence of any air-borne challenge compared with the norm. These relationships may equally fit a non-linear regression where the more extreme levels of disease have disproportionate effects, for example where plant defenses are triggered above certain inoculum thresholds resulting in a cost and risk to plant fecundity. Such a novel approach to identifying and characterizing tolerance using more extreme factor parameter values in evaluation environments should facilitate identification of key traits affecting tolerance, especially in crop mixtures where the dynamics are otherwise too complex to do so by more mechanistic means.

## Conclusions

Given the complexity of the interactions in mixtures and the effects of modifiers on expression of tolerance, applying concepts developed for monocultures to mixtures may not identify the traits responsible. It may be better to consider resilience of the system as a whole and not to adopt only the reductionist approach of trying to improve tolerance through trait selection in monocultures. Resilience would encompass restricting disease development and enhancing yield stability of the mixture rather than focusing on the tolerance traits of individual components.

Whether a crop mixture is more tolerant than a monoculture is the outcome of many plant and microbe community dynamic responses operating under a range of biotic and abiotic challenges. Such variable conditions are a normal part of the environment and required for normal plant development, but it is the extremes conditions, both high and low, that reveal the traits most influential on plant community tolerance. It is unlikely therefore that tolerance can be assessed or selected under normal field trial conditions where treatments tend toward the optimal. Furthermore, it is on-farm performance where conditions are more often sub-optimal where tolerance can be best exploited and therefore where the traits most favored need to be identified and optimized.

## Author contributions

The author confirms being the main contributor of this work and approved it for publication.

## Funding

I am grateful also for financial support for this work from the Rural and Environment Science and Analytical Services (RESAS) Division of the Scottish Government (2011–2016) under its Environmental Change and Food, Land and People Research Programmes.

### Conflict of interest statement

The author declares that the research was conducted in the absence of any commercial or financial relationships that could be construed as a potential conflict of interest.
